# Compartmental and COMSOL Multiphysics 3D Modeling of Drug Diffusion to the Vitreous Following the Administration of a Sustained-Release Drug Delivery System

**DOI:** 10.3390/pharmaceutics13111862

**Published:** 2021-11-04

**Authors:** Emily Dosmar, Gabrielle Vuotto, Xingqi Su, Emily Roberts, Abigail Lannoy, Garet J. Bailey, William F. Mieler, Jennifer J. Kang-Mieler

**Affiliations:** 1 Department of Biology and Biomedical Engineering, Rose-Hulman Institute of Technology, 5500 Wabash Avenue, Terre Haute, IN 47803, USA; vuottogr@rose-hulman.edu (G.V.); sux@rose-hulman.edu (X.S.); evanhavel@kumc.edu (E.R.); brownal2@rose-hulman.edu (A.L.); baileygj@rose-hulman.edu (G.J.B.); 2 Department of Biomedical Engineering, Illinois Institute of Technology, 10 W 35th St., Chicago, IL 60616, USA; wmieler@uic.edu; 3 Department of Ophthalmology and Visual Sciences, University of Illinois at Chicago, 1200 W Harrison St., Chicago, IL 60607, USA; jkangmie@iit.edu

**Keywords:** targeted drug delivery, ocular drug delivery, compartmental modeling, pharmacokinetic modeling, COMSOL 3D modeling, hydrogels, topical delivery, subconjunctival delivery, intravitreal delivery, subretinal delivery

## Abstract

The purpose of this study was to examine antibiotic drug transport from a hydrogel drug delivery system (DDS) using a computational model and a 3D model of the eye. Hydrogel DDSs loaded with vancomycin (VAN) were synthesized and release behavior was characterized in vitro. Four different compartmental and four COMSOL models of the eye were developed to describe transport into the vitreous originating from a DDS placed topically, in the subconjunctiva, subretinally, and intravitreally. The concentration of the simulated DDS was assumed to be the initial concentration of the hydrogel DDS. The simulation was executed over 1500 and 100 h for the compartmental and COMSOL models, respectively. Based on the MATLAB model, topical, subconjunctival, subretinal and vitreous administration took most (~500 h to least (0 h) amount of time to reach peak concentrations in the vitreous, respectively. All routes successfully achieved therapeutic levels of drug (0.007 mg/mL) in the vitreous. These models predict the relative build-up of drug in the vitreous following DDS administration in four different points of origin in the eye. Our model may eventually be used to explore the minimum loading dose of drug required in our DDS leading to reduced drug use and waste.

## 1. Introduction

### 1.1. Ocular Drug Delivery

Drug delivery to the posterior segment is essential to treat chronic ocular diseases such as glaucoma, choroidal neovascularization and secondary age-related macular degeneration [1]. Diabetic macular edema, retinal vein occlusions, and endophthalmitis, when left untreated, can lead to severe visual complications and even blindness [1]. Posterior penetration of antibiotics for the prevention of infections such as endophthalmitis is particularly crucial to the continued success of ophthalmological surgical advancements.

There are four primary routes through which pharmacologic agents can directly enter the eye: topically, from the subconjunctival, through the subretina, and intravitreally. The selection for the most ideal approach depends on a variety of factors including the molecular properties and target tissues of the delivered drug, the frequency of required dosing, and the expected mode of transport for the drug [2]. Each delivery route has its own advantages and disadvantages [1].

Topical delivery accounts for nearly 90% of all ocular drug delivery [3,4]. A major challenge associated with topical drug delivery is pre-ocular retention [5]. Commercial eye drop dispensers commonly deposit a 50 μL volumetric dose, which overwhelms the existing 7 μL of tear fluid resulting in the loss of the majority of drug upon application [5]. Continual tear film turnover is another effective mechanism of clearance for the eye, whereby lacrimation and drainage occurs at ~1 μL/min, or ~16% turnover of tear film per minute [4,5]. Topical delivery is also largely limited by drug absorption and transport across the cornea, which is inefficient due to its small surface area and impermeable characteristics [4,5]. Consequently, intraocular bioavailability of topically supplied solutions is typically <5% of the original dosage amount [4,5,6].

Subconjunctival delivery offers a lower risk of retinal detachment than intravitreal administration, eliminates the issue of patient compliance required by topical delivery, and provides a more adequate drug concentration to target sites than systemic injections [2,7]. An advantage of subconjunctival injection is that the delivered drug must penetrate the sclera, whose permeability is greater than that of the cornea [8,9]. Additionally, and unlike the cornea and conjunctiva, scleral permeability is not dependent on drug lipophilicity [8]. Souli et al. (2001) administered a 20 mg subconjunctival injection of vancomycin (VAN) and after five hours observed a peak concentration of 24.82 ± 3.55 μg/mL in the human aqueous humor, followed by a rapid decline [10]. Similarly, after 6 h following a 20 mg subconjunctival injection, gentamicin levels peaked at ~5 μg/mL in the rabbit vitreous humor [7]. It is suggested that the immediate decrease in detected drug following its peak results from rapid clearance by the aqueous humor [10], whose turnover is about 2.4 ± 0.6 μL/min (250 μL total volume) [11]. These findings indicate that antibiotic drug delivery through subconjunctival injection is achievable; however, a continuous supply of drug is necessary to maintain a therapeutic concentration in the vitreous. 

Intravitreal injection provides a direct route of access for drugs that require entry to the vitreous and have been shown to also better enable drug access to the retina, maximize therapeutic concentrations, while also minimizing the potential for systemic toxicity. Intravitreal injection has been shown to be appropriate for a wide range of therapeutics including low molecular mass drugs and macromolecules such as oligonucleotides and monoclonal antibodies or antibody fragments [12]. However, the half-life of drugs in the vitreous is relatively short, which necessitates repeated injection in many cases [12]. Although low in frequency, it can lead to serious complications such as endophthalmitis, retinal detachment, intravitreal hemorrhage, and cataract [13].

Subretinal injection is considered a more recent and novel approach to ocular drug delivery, has been shown to provide a safe approach to injectable gene therapies [14], and is particularly useful for providing a minimally invasive yet direct route to a very precise location [15]. Typically, a volume of approximately 150 µL is injected, which leads to a transient detachment between the two layers of tissue [16]. A lower drug dose is needed to accurately reach the cells of the subretinal space [15].

### 1.2. Drug Delivery Systems

To combat some of the primary challenges associated with ocular drug delivery, namely, drug loss and the need for continuous dosing, sustained-release drug delivery systems (DDSs) have been developed. These devices are typically an implantable or injectable polymeric housing containing the drug in question that can deliver an effective dose for the required period. DDSs allow for a targeted and continuous supply of drug and show promise as a more effective treatment tool than injection. 

Hydrogels have often been sought after as a tool for DDSs. Hydrogels are polymeric networks capable of retaining absorbed water while maintaining their three-dimensional structures [17,18]. Poly(ethylene glycol) (PEG)-based hydrogels are attractive for use in a variety of biomedical applications due to their highly biocompatible nature and tunable mechanical and degradable properties [19]. The ease of incorporating cells, proteins, and pharmacological agents into hydrogels without interfering with drug efficacy has led to their wide use as drug delivery vehicles [17,18,19,20,21]. When hydrogels are made to degrade hydrolytically, the need for a post-regimen removal surgery is eliminated [17]. Degradation of PEG-based hydrogels is achieved through the utilization of block-copolymer components, such as poly (l-lactic acid) (PLLA), a well-documented hydrolytically degradable hydrophobic polymer [19,20,21,22].

For applications where minimally invasive procedures are desired, including intravitreal injections, thermo-responsive, poly(*N*-isopropylacrylamide) (PNIPAAm) based hydrogels have been used. A unique feature of such hydrogels that make them an attractive option, is that they can be manipulated to have a fluid-like consistency at room temperature and transform into a viscoelastic solid state when they reach physiological temperatures. Due to their fluid-like properties at room temperature, such hydrogels can be injected through 28G needles [13]. The transition temperature of these hydrogels can be modified through cross-linking with increased concentrations of Poly(ethylene glycol) diacrylate (PEG-DA). It has been shown that the transition temperature can be controlled between 32 and 37 °C. These hydrogels may be made fully biodegradable through the incorporation of PEG-PLLA-DA and glutathione as a chain transfer agent [23]. Biodegradability has been shown not to have any effect on the hydrogel transition temperature [23].

### 1.3. Modeling Pharmacokinetics

Ocular drug pharmacokinetic analysis is a powerful tool to predict drug dispersion from its delivery site and to anticipate therapeutic dosing levels. Lee and Robinson developed a simple compartmental model to explore the various aspects of ocular drug penetration pathways by examining mechanisms of clearance, rate constants, and drug solubility [24,25,26,27]. The model describes drug penetration into the vitreous cavity following a subconjunctival injection and demonstrates that a direct pathway through the underlying tissues (e.g., sclera, choroid, and retina) is dominant to indirect penetration through the aqueous chamber or drug absorption into the general circulation [27]. Additionally, the model validated in vivo the dominance of the direct penetration pathway by demonstrating the minimal contribution of indirect and systemic pathways to drug concentrations in the vitreous [24,25,26,27]. 

The primary aim of this study is to develop a model of drug flow through the posterior ocular tissues following topical, subconjunctival, subretinal, and intravitreal drug administration of an injectable vancomycin (VAN)-loaded DDS. We seek to model drug transport from the proposed system to the vitreous and predict the relative time needed to achieve a therapeutic concentration. Using rate constants specific to small molecules and a drug input function derived from our DDS, we aim to predict the minimum DDS drug loading that will result in adequate drug levels in the target tissues. 

## 2. Materials and Methods

Unless otherwise noted, all chemicals were purchased from Sigma Aldrich (St. Louis, MO, USA).

### 2.1. Hydrogel Synthesis

The PNIPAAm-PEG-DA hydrogels were prepared according to a method described and characterized by Kang-Mieler et al. [28,29] and Drapala et al. [23,30]. Briefly, hydrogels were synthesized by dissolving PEG-DA (2 mM), *N*-tert-butylacrylamide (47 mM), and ammonium persulfate (13 mM) in 1× Dulbecco’s Phosphate Buffered Saline (DPBS) (pH 7.4). NIPAAm (350 mM) was subsequently added to create the hydrogel precursor in a 2cc microcentrifuge tube and maintained on ice. 60 mg of VAN was added to the DDS by dissolving it in the precursor. *N*,*N*,*N*’,*N*’-Tetramethylethylenediamine (168 mM) was added to initiate hydrogel polymerization. The procedure described uses free radical polymerization that was left to proceed on ice for a duration of 30 min. Following polymerization, the newly formed hydrogels were collected and washed five times in ddH_2_O. Hydrogels were made in triplicate. 

### 2.2. Effect of Glutathione on Thermo-Responsive Hydrogel Degradation

Thermo-responsive hydrogels were synthesized using the aforementioned technique. Instead of PEG-DA as used above, 2 mM PEG-PLLA-DA (synthesized in lab) was used and glutathione (purity ≥ 98%) was added at different concentrations (from 0, 1.0, and 1.5 mg/mL). Hydrogels were submerged in (4-(2-hydroxyethyl)-1-piperazineethanesulfonicacid) (HEPES) buffer and weighed daily to quantify degradation [31].

### 2.3. Hydrogel Encapsulation Efficiency

The encapsulation efficiency (EE) is an indicator of the initial amount of drug entrapped within the DDS. While classically, this metric is determined by dissolving the DDS to reveal the exact amount of drug trapped inside, in this case, all methods of polymer dissolving resulted in damage to VAN as well. In accordance with the methods reported by Honary et al., the EE of each DDS was determined indirectly by subtracting the quantity of drug lost to the washing phases from the total drug used for encapsulation [32,33]. Drug quantity in the wash samples was determined using a NanoDrop™ 2000/2000C Spectrophotometer (E1% 40, 280 nm) (ThermoFisher Scientific, Grand Island, NY, USA).

### 2.4. Hydrogel Release Profiles

A single (1 mL) hydrogel was placed in 1 mL of Phosphate Buffered Saline (PBS) under static conditions at 37 °C. At predetermined intervals, 1 mL of aqueous media was removed via pipetting and replaced with an equal volume of fresh buffer. VAN concentration in the release samples was quantified using a NanoDrop™ 2000/2000C Spectrophotometer (E1% 40, 280 nm). Cumulative release was calculated relative to EE. The initial burst (IB) was defined as the drug released within the first 5, 12, and 24 h. Release profiles were conducted for 504 h (3 weeks). All release profiles were performed in triplicate. 

**Figure 1:**
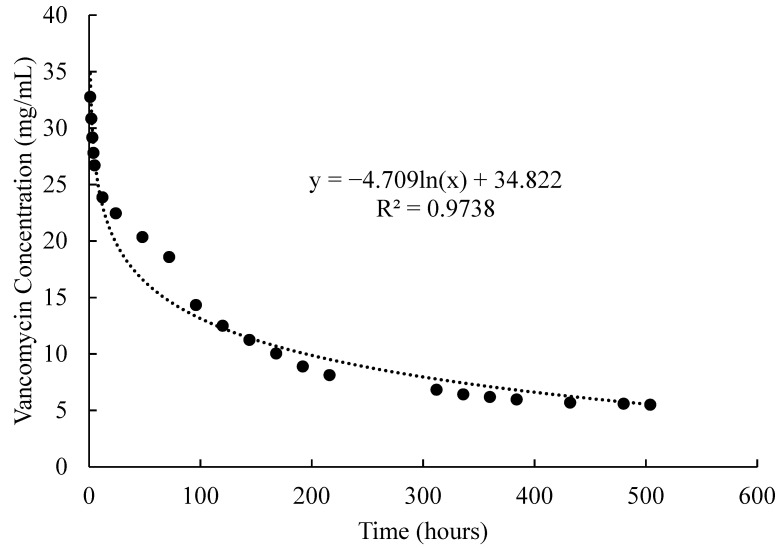
Vancomycin concentration over time as it diffuses out of the PNIPAAm-PEG-DA hydrogel. An equation of the line was fitted to the data and the equation was extrapolated to describe the change in hydrogel concentration over time for the model described.

The cumulative release of VAN from a 1 mL non-degradable PNIPAAm-PEG-DA thermo-responsive hydrogel containing ~34 mg of VAN following the wash cycle (described previously) was considered in terms of hydrogel concentration change over time as seen in Figure 1. A logarithmic curve was fit to the concentration data and an equation to describe the behavior was generated using Microsoft Excel Software Version 2017 (Equation (1)). 

The equation generated to describe the change in hydrogel drug concentration over time can be seen in Equation (1). This equation was used as the *C_H_*(*t*) input function.

(1) $$C_{H}=-4.709\ln\left(t\right)+34.822$$

### 2.5. Compartmental Model

Four different compartmental models of the eye were developed to describe transport into the vitreous originating topically, from the subconjunctival, subretinally, and intravitreally. Each of these models was used to describe drug diffusion from the point of origin to the vitreous while considering drug loss and exchange between each compartment. Experimental cumulative release data of VAN from the DDS were used as the input function (Equation (1)). Rate constants were selected based on an extensive literature review and the surface area of all compartments were assumed to be equal (1 cm^2^). The potential contributions of lateral diffusion or convective flows in the choriocapillaris were not considered as part of this model [34]. The physical and anatomical barriers that were considered were analyzed in series with each barrier (or compartment) allowing a fraction of the contained drug (C) to pass through to the subsequent tissue [34]. Additionally, it is assumed that there is no metabolism of the drug in the sclera or retina and hence, the bioavailability remains constant throughout [34]. It is assumed that backflow between compartments was negligible [34]. The model was simulated using MATLAB version R2019a software.

Rate constants were selected based on an extensive literature review. The sclera is permeable to hydrophilic compounds [35,36,37,38]. Permeability to the retinal pigment epithelium is 1–2 orders of magnitude slower than in the sclera [39]. Surprisingly, experiments have shown that the choroidal blood flow has far less contribution to the retinal and subconjunctival drug concentrations than previously thought [40,41,42]. Alternatively, many researchers continue to consider the choroidal blood flow as a substantial obstacle to successful drug penetration into the retina [43]. Scenarios with and without high choroidal blood flow contributions were considered. The rate constants describing backflow between compartments were considered to be negligible and therefore set to zero (0) [34]. 

Thresholds: We have previously determined, empirically, the minimum therapeutic dose for VAN [44]. Considering a vitreous size of 1 cm^2^, 0.007 mg/mL is considered a therapeutic does to kill bacteria on the total surface area [44]. A study completed by Souli et al. (2001) measured a peak VAN concentration of 24.82 µg/mL ± 3.55 µg/mL in the human aqueous humor following bolus subconjunctival injections [10]. Therefore, 0.024 mg/mL was also tracked and considered as a threshold in this model. While it is not considered the minimum required dose for our system, it is a goal that the value also be achieved within the first 24 h of release. 

### 2.6. COMSOL Multiphysics Model 

COMSOL Multiphysics was used to simulate the drug diffusion profile to the vitreous from each point of origin, where a “Time-Dependent” “Transport of Diluted Species in a Porous Media” study was constructed. The model was created by establishing three 2D work planes for each tissue layer in the eye based on each compartmental model. In each work plane, a curve with a diameter and thickness of the tissue layer was created and revolved around the central axis. After the three layers were constructed, a solid sphere with the diameter of the vitreous was created to represent the vitreous humor. A structure with the geometry of a standard contact lens was created to simulate a point of origin for the DDS. An additional layer revolving around this structure was created to simulate the rate of loss from the drug site and a second layer around the vitreous to stimulate the vitreal drug loss. The COMSOL “Form Union” method was employed to create a single geometry object composed of many different domains. Material properties (coefficient of thermal expansion, bulk viscosity, dynamic viscosity, density, etc.) and transport properties (drug permeation rate for each compartment), were applied to each layer. The initial concentration for the compartments were assumed to be zero. An inflow to the contact structure was created as an input function of the cumulative release of VAN from DDS. The concentration of simulated contact lens was assumed to be the initial concentration of a drug delivery system implanted in the space of origin in units mol/m^3^. Outflows of the corresponding concentration were added to each of the elimination layers. After all the parameters were set, the element size in Mesh was chosen to be “Normal” to discretize the geometry for current simulation. The simulation was executed over 100 h and the data were collected at 10 h increments.

### 2.7. Statistical Analysis

All empirically derived values are reported as the mean ± standard deviation and in all graphs, error bars represent standard deviation. All statistical differences were determined using one-way ANOVA testing and unless otherwise noted, significance represents *p* ≤ 0.05. 

## 3. Results

### 3.1. In Vitro Drug Delivery System Results

**Figure 2:**
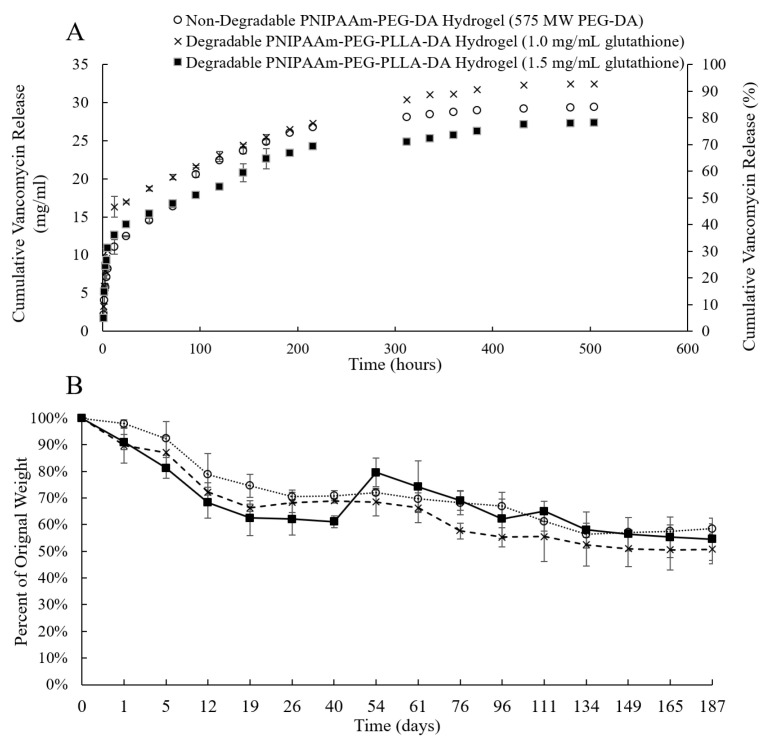
The effects of glutathione on release and degradation of 1 mL thermo-responsive PNIPAAm-PEG-DA hydrogel DDS. (**A**) Release from a 1 mL thermo-responsive PNIPAAm-PEG-DA-based hydrogel and hydrogels containing PEG-PLLA-DA and 1.0 and 1.5 mg/mL glutathione, respectively. Non-degradable hydrogels showed a significantly lower (*p* < 0.05) initial burst than both biodegradable hydrogels at 5, 12 and 24 h. (**B**) Hydrogel degradation over time for the non-degradable, 1.0 mg/mL and 1.5 mg/mL hydrogels over 187 days.

An amount of 34 mg of VAN was successfully encapsulated into 1 mL PNIPAAm-PEG-DA thermo-responsive hydrogels following washing (data previously published [44]), yielding an EE of 57%. The initial VAN releases from these hydrogels were 23% ± 0.1, 31% ± 0.95 and 36% ± 0.06 at 6, 12 and 24 h, respectively (Figure 2A). VAN release continued at a steady rate (~1.5 mg/mL) for two weeks until finally tapering off and plateauing at 84% ± 0.08 cumulative release at 504 h (21 days) (Figure 2A). 

The VAN release from 1 mL non-degradable PNIPAAm-PEG-DA hydrogels compared to hydrogels containing PEG-PLLA-DA and 1.0 mg/mL and 1.5 mg/mL glutathione, respectively, can be found in Figure 2A. Non-degradable hydrogels cumulatively released more than 2 mg/mL (*p* < 0.05) VAN than both biodegradable hydrogels at 5, 12 and 24 h. Following 48 h and until the conclusion of this study, the non-degradable hydrogels consistently released statistically more VAN than those containing 1.5 mg/mL glutathione but statistically less than those containing 1.0 mg/mL glutathione. In total, the non-degradable hydrogels released 29.4 ± 0.08 mg/mL VAN, while the hydrogels containing 1.0 mg/mL and 1.5 mg/mL glutathione 32.5 ± 0.05 mg/mL and 27.4 ± 0.03 mg/mL, respectively.

Degradability in terms of percent weight loss was examined for hydrogels containing PEG-PLLA-DA and glutathione and compared to non-degradable hydrogels. At 40 days, the 1.5 mg/mL glutathione hydrogel was statistically different (*p* < 0.05) than the 1.0 mg/mL glutathione and non-degradable hydrogels (61.1% ± 2.24 of original weight compared to 68.9% ± 0.7 and 70.8% ± 1.89 for the 1.0 mg/mL and non-degradable hydrogels, respectively). At 54 days, the 1.0 mg/mL and 1.5 mg/mL glutathione hydrogels were statistically significant than each other (68.5% ± 5.15 and 79.6% ± 5.42 of original weight, respectively) and at 76 days the 1.0 mg/mL glutathione hydrogels were statistically different than the non-degradable and the 1.5 mg/mL glutathione hydrogels (57.7% ± 2.97 of original weight compared to 69% ± 3.64 for both the 1.0 mg/mL glutathione and non-degradable hydrogels). Overall, all three of the hydrogels lost at least 40% of their original weight by the time this study concluded at 187 days (Figure 2B). The increased weight (or “weight gain”) that is seen between 40 and 54 days for the 1.5 mg/mL glutathione hydrogel may be due to an increased uptake in water (swelling). It did not appear that incorporating glutathione into these hydrogels significantly impacted the weight lost in the first 187 days measured. 

### 3.2. Compartmental Model

**Figure 3:**
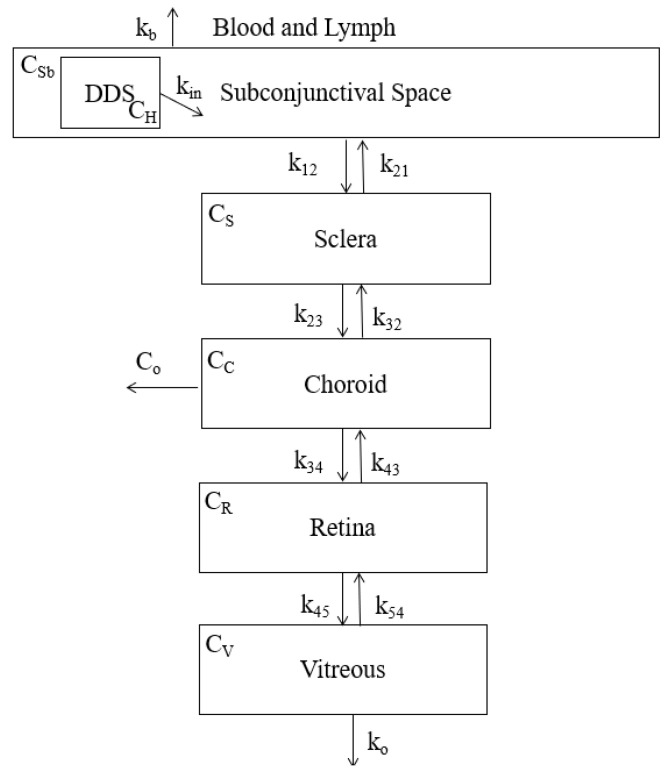
Compartment model that considers the layers of tissues that a drug must transverse in order to enter the vitreous chamber following a subconjunctival entry point. The encapsulated drug flows out of the DDS into the subconjunctival space and must pass through the sclera, choroid, and retina before finally reaching the vitreous. Drug loss to the blood and lymph is also considered *k_b_*. *C_H_*, *C_Sb_*, *C_S_*, *C_C_*, *C_R_*, and *C_V_* represent the drug concentration in their respective compartments. All values of k represent the rate of drug flow into and out of their respective compartments. *C_o_* represents the fraction of drug lost from the choroid.

The compartmental model for the subconjunctival entry route is shown below in Figure 3. The release from the DDS is shown as the input of drug into the subconjunctival. The boxed compartments represent the path of drug to the vitreous. The equations generated from the model are shown below the figure. 

The equations generated from the subconjunctival model shown in Figure 3 are shown below in Equations ((2)–(6)).

(2) $$\frac{dC_{Sb}}{dt}=k_{in}\left(-4.709\log\left(t\right)+34\right)-(k_{12}+k_{b})C_{sb}+k_{21}C_{S}$$

(3) $$\frac{dC_{S}}{dt}=k_{12}C_{Sb}+k_{32}C_{C}-\left(k_{21}+k_{23}\right)C_{S}$$

(4) $$\frac{dC_{C}}{dt}=k_{23}C_{S}+k_{43}C_{R}-\left(k_{32}+k_{34}\right)C_{C}-C_{o}C_{C}$$

(5) $$\frac{dC_{R}}{dt}=k_{34}C_{C}+k_{54}C_{V}-\left(k_{43}+k_{45}\right)C_{R}$$

(6) $$\frac{dC_{V}}{dt}=k_{45}C_{R}-\left(k_{54}+k_{o}\right)C_{V}$$

where

$C_{Sb}$ is the concentration of drug in the subconjunctival space,

$C_{S}$ is the concentration of drug in the sclera,

$C_{C}$ is the concentration of drug in the choroid,

$C_{R}$ is the concentration of drug in the retina,

$C_{V}$ is the concentration of drug in the vitreous,

$k_{in}$ is the rate of drug flowing from the DDS into the subconjunctival space,

$k_{b}$ is the rate of drug flowing out into the blood,

$k_{12}$ is the rate of drug flowing from the subconjunctival space to the sclera,

$k_{21}$ is the rate of drug flowing from the sclera to the subconjunctival space,

$k_{23}$ is the rate of drug flowing from the sclera to the choroid,

$k_{32}$ is the rate of drug flowing from the choroid to the sclera,

$k_{34}$ is the rate of drug flowing from the choroid to the retina,

$k_{43}$ is the rate of drug flowing from the retina to the choroid,

$k_{45}$ is the rate of drug flowing from the retina to the vitreous,

$k_{54}$ is the rate of drug flowing from the vitreous to the retina,

$k_{O}$ is the rate of drug flowing out of the vitreous, 

and $C_{o}$ is the rate of the drug leaving the choroid. 

All *k* values are in units hr^−1^.

**Figure 4:**
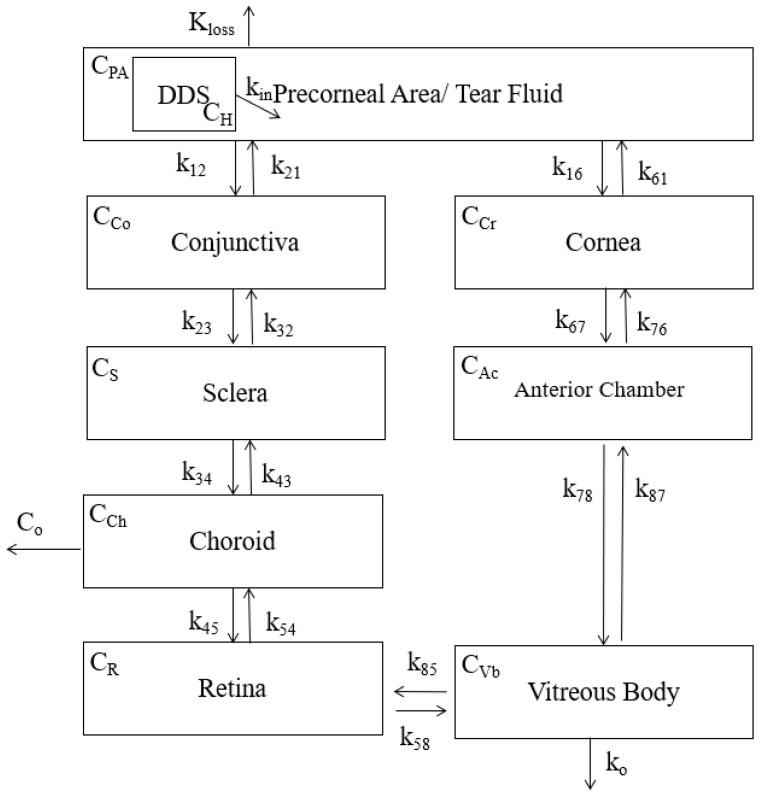
Compartment model that considers the layers of tissues that a drug must transverse in order to enter the vitreous chamber following topical entry. The encapsulated drug flows out of the DDS into the precorneal area where it mixes with the tear fluid. From there, it can pass either through the cornea and the anterior chamber or alternatively, through the conjunctiva, sclera, choroid, and retina before finally reaching the vitreous. Drug loss (*K_loss_*) due to fluid runoff from the eye is also considered. *C_H_*, *C_PA_*, *C_Co_*, *C_S_*, *C_Ch_*, *C_R_*, *C_Cr_*, *C_Ac_* and *C_Vb_* represent the drug concentration in their respective compartments. $C_{o}$ is the fraction of drug lost from the choroid. All values of *k* represent the rate of drug flow into and out of their respective compartments.

The compartmental model for the topical entry route is shown below in Figure 4. The release from the DDS is shown as the input of drug into the precorneal area. The boxed compartments represent the path of drug to the vitreous. The equations generated from the model are shown below the figure. 

The equations generated from the topical model shown in Figure 4 are shown below in Equations (7)–(14).

(7) $$\frac{dC_{PA}}{dt}=k_{in}\left(-4.709\log\left(t\right)+34\right)-(k_{12}+k_{loss})C_{PA}+k_{21}C_{Co}$$

(8) $$\frac{dC_{Co}}{dt}=k_{12}C_{PA}+k_{32}C_{S}-\left(k_{21}+k_{23}\right)C_{Co}$$

(9) $$\frac{dC_{S}}{dt}=k_{23}C_{Co}+k_{43}C_{Ch}-\left(k_{32}+k_{34}\right)C_{S}$$

(10) $$\frac{dC_{Ch}}{dt}=k_{34}C_{S}+k_{54}C_{R}-\left(k_{43}+k_{45}\right)C_{Ch}-C_{o}$$

(11) $$\frac{dC_{R}}{dt}=k_{45}C_{Ch}-\left(k_{54}+k_{85}\right)C_{R}$$

(12) $$\frac{dC_{Cr}}{dt}=k_{16}C_{Ac}+k_{58}C_{R}-\left(k_{61}+k_{67}\right)C_{Cr}$$

(13) $$\frac{dC_{Ac}}{dt}=k_{67}C_{Cr}+k_{87}C_{Vb}-\left(k_{76}+k_{78}\right)C_{Ac}$$

(14) $$\frac{dC_{Vb}}{dt}=k_{78}C_{S}+k_{54}C_{R}-\left(k_{87}+k_{85}+k_{o}\right)C_{Vb}$$

where

$C_{PA}$ is the concentration of drug in the precorneal area/tear fluid,

$C_{Co}$ is the concentration of drug in the conjunctiva,

$C_{S}$ is the concentration of drug in the sclera,

$C_{Ch}$ is the concentration of drug in the choroid,

$C_{o}$ is the fraction of drug lost from the choroid,

$C_{Cr}$ is the concentration of drug in the cornea,

$C_{Ac}$ is the concentration of drug in the anterior chamber,

$C_{Vb}$ is the concentration of drug in the vitreous body,

$k_{in}$ is the rate of drug flowing from the DDS into the precorneal area/tear fluid,

$k_{loss}$ is the rate of drug flowing out of the eye,

$k_{12}$ is the rate of drug flowing from the precorneal area to the conjunctiva,

$k_{21}$ is the rate of drug flowing from the conjunctiva to the precorneal area,

$k_{23}$ is the rate of drug flowing from the conjunctiva to the sclera,

$k_{32}$ is the rate of drug flowing from the sclera to the conjunctiva,

$k_{34}$ is the rate of drug flowing from the sclera to the choroid,

$k_{43}$ is the rate of drug flowing from the choroid to the sclera,

$k_{45}$ is the rate of drug flowing from the choroid to the retina,

$k_{54}$ is the rate of drug flowing from the retina to the choroid,

$k_{16}$ is the rate of drug flowing from the precorneal area to the cornea,

$k_{61}$ is the rate of drug flowing from the cornea to the precorneal area,

$k_{67}$ is the rate of drug flowing from the cornea to the anterior chamber,

$k_{76}$ is the rate of drug flowing from the anterior chamber to the cornea,

$k_{78}$ is the rate of drug flowing from the anterior chamber to the vitreous,

$k_{87}$ is the rate of drug flowing from the vitreous to the anterior chamber,

$k_{58}$ is the rate of drug flowing from the retina to the vitreous,

$k_{85}$ is the rate of drug flowing from the vitreous to the retina,

and

$k_{O}$ is the rate of drug flowing out of the vitreous.

All *k* values are in units hr^−1^.

**Figure 5:**
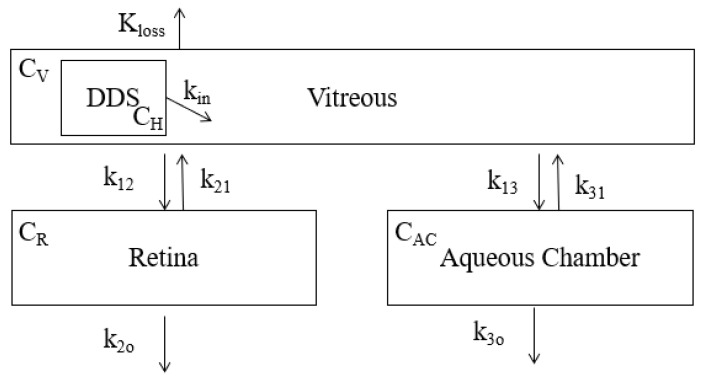
Compartment model that considers the layers of tissues that a drug must transverse in order to enter the vitreous chamber following an intravitreal entry point. The encapsulated drug flows out of the DDS directly into the vitreous. From there, drug exchange can occur between the vitreous and the retina and the vitreous and the aqueous chamber. Additional systemic drug loss (*K_loss_*) is also considered. *C_H_*, *C_V_*, *C_R_* and *C_Ac_* represent the drug concentration in their respective compartments. All values of k represent the rate of drug flow into and out of their respective compartments.

The compartmental model for the vitreal entry route is shown below in Figure 5. The release from the DDS is shown as the input of drug into the vitreous. The boxed compartments represent the path of drug from the vitreous. The equations generated from the model are shown below the figure. 

The equations generated from the intravitreal model shown in Figure 5 are shown below in Equations (15)–(17).

(15) $$\frac{dC_{V}}{dt}=k_{in}\left(-4.709\log\left(t\right)+34\right)-(k_{12}+k_{13}+k_{b})C_{V}$$

(16) $$\frac{dC_{R}}{dt}=k_{12}C_{V}-\left(k_{21}+k_{2o}\right)C_{R}$$

(17) $$\frac{dC_{AC}}{dt}=k_{13}C_{V}-\left(k_{31}+k_{3o}\right)C_{AC}$$

where

$C_{V}$ is the concentration of drug in the vitreous,

$C_{R}$ is the concentration of drug in the retina,

$C_{AC}$ is the concentration of drug in the aqueous chamber,

$k_{in}$ is the rate of drug flowing from the DDS into the vitreous,

$k_{loss}$ is the rate of drug lost systemically,

$k_{12}$ is the rate of drug flowing from the vitreous to the retina,

$k_{21}$ is the rate of drug flowing from the retina to the vitreous,

$k_{13}$ is the rate of drug flowing from the vitreous to the aqueous chamber,

$k_{31}$ is the rate of drug flowing from the aqueous chamber to the vitreous,

$k_{2o}$ is the rate of drug flowing out of the retina,

and

$k_{3o}$ is the rate of drug flowing out of the anterior chamber.

All *k* values are in units hr^−1^.

**Figure 6:**
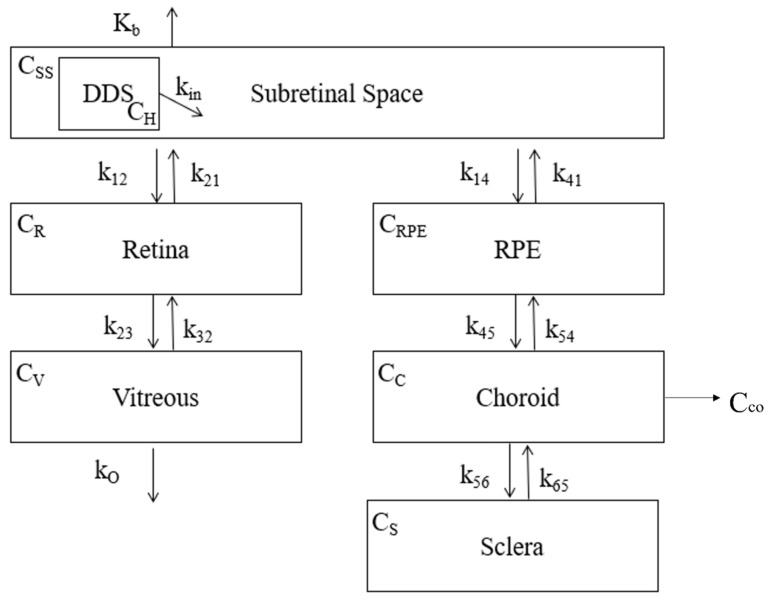
Compartment model that considers the layers of tissues that a drug must transverse in order to enter the vitreous chamber following subretinal entry. The encapsulated drug flows out of the DDS into the subretinal space. From there, it can pass either through the retina into the vitreous or through the retinal pigment epithelium (RPE) and the choroid into the sclera. Drug loss to the blood is also considered (*K_b_*). *C_H_*, *C_SS_*, *C_R_*, *C_V_*, *C_RPE_*, *C_C_* and *C_S_* represent the drug concentration in their respective compartments. *C_o_* represents the fraction of drug lost from the choroid All values of k represent the rate of drug flow into and out of their respective compartments. It should also be noted that loss from the sclera was not considered due to the focus on the vitreous.

The compartmental model for the subretinal entry route is shown below in Figure 6. The release from the DDS is shown as the input of drug into the subretinal space. The boxed compartments represent the path of drug to the vitreous. The equations generated from the model are shown below the figure. 

The equations generated from the subretinal model shown in Figure 6 are shown below in Equations (18)–(23).

(18) $$\frac{dC_{SS}}{dt}=\;k_{in}\left(-4.709\log\left(t\right)+34\right)-(k_{12}+k_{14}+k_{b})C_{SS}$$

(19) $$\frac{dC_{R}}{dt}=\;k_{12}C_{SS}+k_{32}C_{V}-\left(k_{21}+k_{23}\right)C_{R}$$

(20) $$\frac{dC_{V}}{dt}=\;k_{23}C_{R}-\left(k_{32}+k_{o}\right)C_{V}$$

(21) $$\frac{dC_{RPE}}{dt}=\;k_{14}C_{SS}+k_{54}C_{C}-\left(k_{41}+k_{45}\right)C_{RPE}$$

(22) $$\frac{dC_{C}}{dt}=\;k_{45}C_{C}+k_{65}C_{S}-\left(k_{54}+k_{56}\right)C_{C}-C_{co}$$

(23) $$\frac{dC_{S}}{dt}=\;k_{56}C_{C}-k_{65}C_{S}$$

where

$C_{SS}$ is the of drug in the subretinal space,

$C_{R}$ is the concentration of drug in the retina,

$C_{V}$ is the concentration of drug in the vitreous,

$C_{RPE}$ is the concentration of drug in the retinal pigment epithalamium (RPE),

$C_{C}$ is the concentration of drug in the choroid,

$C_{Co}$ is the rate of the drug leaving the choroid,

$C_{S}$ is the concentration of drug in the sclera,

$k_{in}$ is the rate of drug flowing from the DDS into the subretinal space,

$k_{b}$ is the rate of drug flowing out into the blood,

$k_{12}$ is the rate of drug flowing from the subretinal space to the retina,

$k_{21}$ is the rate of drug flowing from the retina to the subretinal space,

$k_{23}$ is the rate of drug flowing from the retina to the vitreous,

$k_{32}$ is the rate of drug flowing from the vitreous to the retina,

$k_{14}$ is the rate of drug flowing from the subretinal space to the RPE,

$k_{41}$ is the rate of drug flowing from the RPE to the subretinal space,

$k_{45}$ is the rate of drug flowing from the RPE to the choroid,

$k_{54}$ is the rate of drug flowing from the choroid to the RPE,

$k_{56}$ is the rate of drug flowing from the choroid to the sclera,

$k_{65}$ is the rate of drug flowing from the sclera to the choroid,

and

$k_{O}$ is the rate of drug flowing out of the vitreous.

All *k* values are in units hr^−1^.

Based on values gleaned from the literature, the non-zero rate constant values were set as found in Table 1.

**Figure 7:**
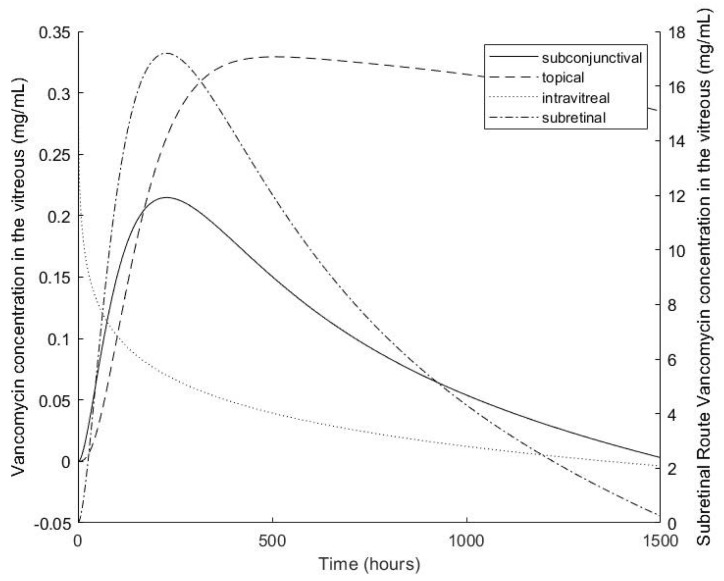
Vancomycin concentration in the vitreous over 1500 h as predicted by compartment models of a DDS containing drug originating topically, subconjunctivally, intravitreally and subretinally and simulated used MATLAB version R2019a software.

The results of a simulation of each of the four models over 1500 h are shown in Figure 7. Based on the model predictions, topical, subconjunctival, subretinal, and intravitreal administration took most (~500 h to least (0 h) amount of time to reach peak concentrations in the vitreous, respectively. From the topical, subconjunctival, subretinal, and intravitreal points of origin it took ~29, ~12, ~1 and 0 h to achieve 0.007 mg/mL VAN dosing levels in the vitreous, respectively. Based on these simulations, the initial intravitreal concentration was the highest at ~0.34 mg/mL with direct entry into the intravitreal space, but rapidly fell as concentrations from the subconjunctival, subretinal and topical simulations rose and peaked at ~0.22, ~0.34 and ~0.33 mg/mL, respectively. The intravitreal concentration with the topical simulation was maintained for the longest of the four models.

### 3.3. COMSOL Multiphysics Model 

**Figure 8:**
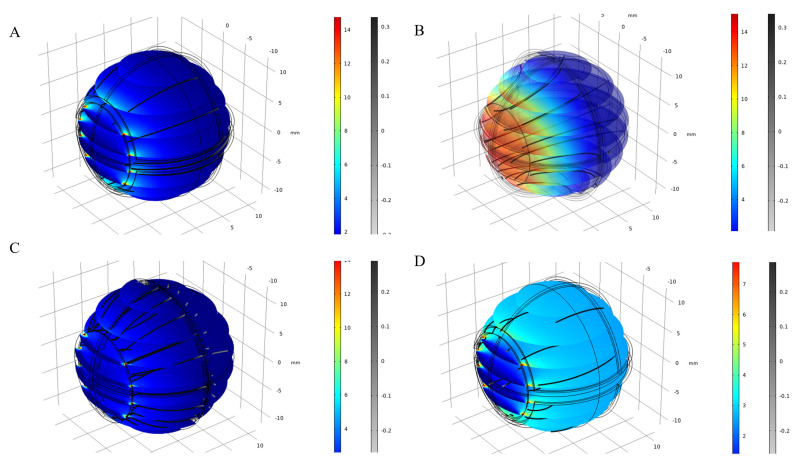
COMSOL Multiphysics Model simulation of drug entry routes originating from (**A**) the subconjunctival, (**B**) topically, (**C**) the vitreous and (**D**) the subretina and penetrating into the vitreous. The concentration profile is demonstrated using both streamline and slices. The right color bar represents the concentration spectrum for the streamlines and the left represents the slices.

The COMSOL models for each of the four entry routes are shown below in Figure 8. The release from the DDS is shown as the input of drug into the location in question. The concentration profile is illustrated as both Streamline (with slices) and Surface. These models predict the relative build-up of drug in the vitreous following DDS administration in four different points of origin in the eye.

**Figure 9:**
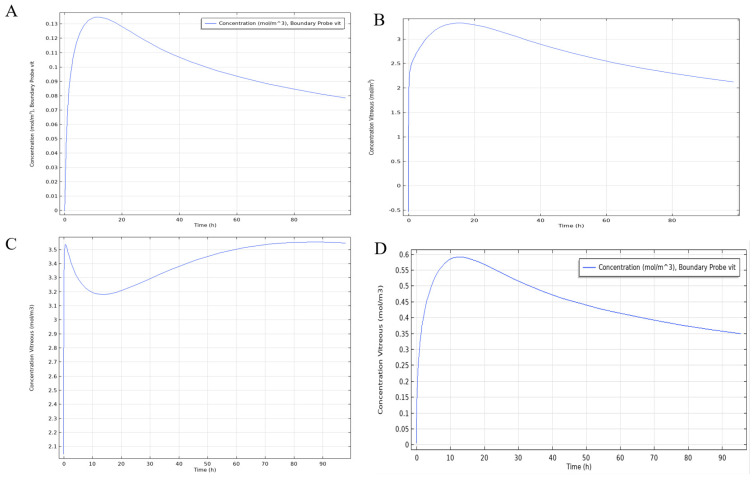
COMSOL Multiphysics 3D Model simulation of drug concentrations in the vitreous following entry routes originating from (**A**) the subconjunctival, (**B**) topically, (**C**) the vitreous and (**D**) the subretina; and penetrating into the vitreous.

Final results from the COMSOL 3D model simulation were displayed as a diffusion profile over a period of 100 h (Figure 9). According to this model series, the subconjunctival, topical, and subretinal delivery routes peaked at approximately ~10 h at ~0.135, ~3.75 and ~0.59 mol/m^3^, respectively. The intravitreal delivery route showed an almost immediate concentration of ~3.6 mol/m^3^ followed by an initial decline and subsequent increase in concentration that was maintained for the duration of the simulation.

## 4. Discussion

Using a VAN (34 mg, 57% EE) containing DDS with biodegradable potential [23,30] capable of in vivo release for 21 days, we have developed a series of compartmental and 3D COMSOL models able to predict the relative drug penetration over an extended period when placed in four different locations in the eye. To our knowledge, this is the first model that incorporates the release behavior of an experimentally tested sustained-release DDS.

As previously stated, while our empirically determined minimum therapeutic dose for VAN was 0.007 mg/mL, 0.24 mg/mL served as a threshold for optimal dosing for the first 24 h of release. Based on the compartment model predictions, this value was achieved in the vitreous following all routes (~0.34, ~0.34 and ~0.33 mg/mL, for intravitreal, subretinal, and topical routes, respectively) except for the subconjunctival, which fell just short at ~0.22 mg/mL. These thresholds were not achieved within a 24 h time frame; however, minimum therapeutic levels were achieved within 24 h for all four models. It should be noted that in a previous study, this DDS was evaluated in a rodent model and was able to successfully prevent infection in the vitreous within the first 24 h following exposure to *Staphylococcus aureus* bacteria and placement in the subconjunctival space. These results suggest that the minimum therapeutic dose can be and is achieved in vivo [44].

The COMSOL simulation also saw the 0.24 mg/mL (0.17 mol/m^3^) threshold was achieved for the topical, intravitreal, and subretinal delivery routes which peaked at approximately 10 h at ~3.75, ~3.6 and ~0.59 mol/m^3^, respectively. However, like the compartment model, the subconjunctival delivery route fell slightly short at ~0.135 mol/m^3^. The minimum therapeutic dose was achieved for all four routes. Both the compartmental and COMSOL models showed similar behaviors for the subconjunctival and subretinal delivery routes; however, they diverged in the predicted behavior of the topical and intravitreal routes which showed a maintained concentration in the precorneal area with the compartment model but not the COMSOL model and a concentration plateau in the vitreous in the COMSOL model but not the compartment model. The compartment model predicts behavior more in line with what is expected.

Several areas of potential improvement to our models have been identified including incorporating more consistent rate constants and elimination fraction constants as well as considering the diversity in the surface area for each compartment. An even more extensive examination of the various rate constants and their relative effect on model performance could provide greater clarity as to the dominant pathways of ocular drug penetration. While VAN was selected as our model drug, rate constants found in the literature were not all VAN specific, which is an unavoidable limitation. Additionally, VAN is only one of several prophylactic antibiotics used for posterior endophthalmitis prevention [46] and adapting our system for alternative antibiotics could lead to its eventual use in more diverse applications. Furthermore, VAN delivery to an infected and therefore, inflamed eye, could lead to different behaviors of drug penetration throughout the ocular cavity. The degradation study described in this paper showed that our DDS does have biodegradable potential; however, for the sake of simplification, we chose to use VAN release behavior extracted from the non-degradable hydrogel for the model. In the future, we would like to incorporate the behavior from a fully degradable system which would improve the clinical relevance of our model.

The in vivo validation of our model would greatly improve its accuracy and usefulness as a tool for predicting DDS loading dose requirements and overall drug penetration. A previous study exploring the in vivo efficacy of our DDS in a rodent model did yield results consistent with the findings from this study; however, the data are still incomplete [44]. Finally, due to the very nature of compartmental pharmacokinetic modeling, we cannot speculate as to where the drug exactly physically distributes into each compartment. In particular, where subretinal drug delivery is concerned, a precise entry location is crucial. It is therefore, an unfortunate yet unavoidable reality that these models can only provide information regarding drug distribution and rate of drug transfer between the compartments over time.

## 5. Conclusions

These models predict the relative build-up of drug in the vitreous following DDS administration in four different points of origin in the eye. Overall, these models are preliminary but show promise for use in predicting the behavior of small molecule delivery from a DDS placed at various locations on or in the eye. Models such as those described in this paper can be used with various input functions to make clinical predictions without animal subjects and can preliminarily predict the performance of a DDS that had been evaluated in vitro. Our model might eventually be used to explore the minimum loading dose of drug in our DDS required to achieve a therapeutic concentration in the vitreous leading to reduced drug use and overall waste.

## Figures and Tables

**Table 1:** The non-zero rate constants selected for the model *.

Factor	Values (hr^−1^)	Rationale
Drug permeation across the cornea	0.0216	Parameter values implemented in an ocular compartmental absorption and transmit model of dexamethasone [42]
Drug permeation across the conjunctiva	0.0018	Parameter values implemented in an ocular compartmental absorption and transmit model of dexamethasone [42]
Drug permeation across the aqueous chamber	0.0189	Parameter values implemented in an ocular compartmental absorption and transmit model of dexamethasone [42]
Drug permeation across the iris and ciliary	3.6	Parameter values implemented in an ocular compartmental absorption and transmit model of dexamethasone [42]
Drug permeation across the sclera	0.05472	Parameter values implemented in an ocular compartmental absorption and transmit model of dexamethasone [42]
Drug permeation across the choroid	1.782	Parameter values implemented in an ocular compartmental absorption and transmit model of dexamethasone [42]
Drug permeation across the retina	1.782	Parameter values implemented in an ocular compartmental absorption and transmit model of dexamethasone [42]
Drug permeation across the vitreous humor	0.0234	Parameter values implemented in an ocular compartmental absorption and transmit model of dexamethasone [42]
Drug permeation across the RPE	0.000936	Derived values used in modeling the intravitreal pharmacokinetics of antibody fragments [45]
Drug elimination from the vitreous	0.008208	Derived values used in modeling the intravitreal pharmacokinetics of antibody fragments [45]
Drug elimination from systemic absorption	0.0329	Parameter values implemented in am ocular compartmental absorption and transmit model of dexamethasone [42]

* Note that while many of the values are for the drug dexamethasone, it is not expected that physicochemical properties of the drug to impair a model that relies on simple diffusion.

## Data Availability

Not applicable.
